# KG-MACNF: A nonlinear cross-modal fusion model for predicting drug-target interactions via multi-relational embedding and fine-grained structure

**DOI:** 10.1371/journal.pone.0331037

**Published:** 2025-09-09

**Authors:** Yihan Feng, Xixin Yang, Yuanlin Guan, Jinyao Zhang, Hang Yang, Zhongyu Wang, Qi Cheng

**Affiliations:** 1 College of Computer Science and Technology, Qingdao University, Qingdao, Shandong, China; 2 School of Automation, Qingdao University, Qingdao, Shandong, China; 3 School of Mechanical and Automotive Engineering, Qingdao University of Technology, Qingdao, Shandong, China; Xinjiang Technical Institute of Physics and Chemistry, CHINA

## Abstract

Drug-target interaction (DTI) prediction is essential for the development of novel drugs and the repurposing of existing ones. However, when the features of drug and target are applied to biological networks, there is a lack of capturing the relational features of drug-target interactions. And the corresponding multimodal models mainly depend on shallow fusion strategies, which results in suboptimal performance when trying to capture complex interaction relationships. Therefore, this study proposes a novel framework named KG-MACNF. This framework utilizes knowledge graph embedding (KGE) techniques to capture multi-level relational features of entities in large-scale biological networks. Simultaneously, our innovative PoolGAT network, along with CTD descriptors, is employed to extract drug structural features and protein sequence information. Finally, by employing our innovative nonlinear-driven cross-modal attention fusion network, the framework efficiently integrates these multimodal data and generates the final DTI prediction results. Experiments on two publicly available datasets, Yamanishi_08’s and BioKG, demonstrate the substantial advantages of KG-MACNF in DTI prediction. KG-MACNF demonstrates robust stability, especially under imbalanced data conditions. This study successfully overcomes the bottlenecks of prior models in utilizing modality information and feature complementarity, providing a more accurate tool for drug discovery and DTI prediction.

## Introduction

Drug-target interaction (DTI) prediction plays a crucial role in drug discovery and precision medicine, aiming to elucidate the potential interactions between drug molecules and biological targets. By assessing the bioactivity and potential side effects of drugs, it establishes a scientific foundation for personalized treatment strategies [1]. To identify drugs that are both effective and safe for specific proteins, pharmacologists have to screen vast numbers of chemical compounds [2]. However, performing biological experiments is labor-intensive, time-consuming, and costly. As a result, an increasing number of studies have shifted towards computer-aided DTI prediction [3].

Previous in silico methods have primarily focused on learning features from a single modality. Specifically, DTI prediction is performed by extracting fixed-dimensional feature vectors (e.g., molecular fingerprints, protein sequence descriptors) and integrating them with machine learning (ML) models (e.g., support vector machines (SVM), random forests (RF) [4–6]. However, these approaches rely heavily on hand-crafted features and labeled data. Recently, the rise of deep learning (DL) has spurred efforts to automatically learn DTI from low-level representations [7], or to use convolutional neural networks (CNNs) to extract molecular sequence features [8,9]. Other studies have explored deep graph neural networks (GNNs) for DTI prediction, as GNNs naturally represent molecular structures [10,11]. But these methods overlook the principle that similar drugs tend to target similar proteins, a concept that can be applied within the network to guide DTI prediction. Consequently, integrating various similarity sub-networks and known DTI networks has emerged as an effective strategy to enhance prediction performance. iGRLDTI [12] presents a node-dependent local smoothing approach inside a heterogeneous biological information network to improve drug and target representations for DTI prediction. NEDTP [13] further improves prediction performance by incorporating diverse similarity networks and using random walks to extract similarity information from the topological structure. Although network-based approaches exploit topological similarities to improve prediction accuracy, they frequently focus on local node or edge features, ignoring the complex interactions within large-scale biological networks. To address these issues, researchers have started constructing large-scale biological knowledge graphs (KG) and applying knowledge graph embedding (KGE) techniques to integrate multi-source information and capture complex relationships [14,15]. For instance, TriModel [16] proposes a specific KGE model to learn embeddings for both drugs and proteins, transforming the DTI prediction task into a link prediction task within the KG. KG_NFM [17] develops a unified DTI prediction framework by integrating KG with recommendation systems. Although these methods have achieved significant advancements, each retains its inherent strengths and weaknesses. KG-based approaches excel at capturing complex relationships among drugs, targets, and other biological entities within large-scale networks from a macroscopic perspective. However, they frequently overlook the fine-grained modeling of molecular microstructures. In contrast, feature-based methods are adept at extracting fine-grained structural characteristics of individual molecules, allowing for a deeper exploration of molecular details. Despite their respective limitations, the strengths of both approaches complement one another, and it is this complementarity that drives the development of multimodal models.

As a result, an increasing number of studies has focused on integrating multiple feature types, including drug-drug interactions [18] and protein-protein interactions [19]. Specifically, MDTips [20] enhances DTI prediction performance by integrating multiple information sources, including KG, gene expression profiles, and drug/target structural information, thereby enriching the feature set. LG-DTI [21] integrates local features of drugs and proteins with global representations learned via a semi-supervised heterogeneous network embedding, utilizing a RF classifier for DTI prediction. MSI-DTI [22] address the DTI task by employing various feature extractors to obtain multi-angle features from drug/target data. Although these models outperform unimodal approaches, they are limited by information redundancy when dealing with highly heterogeneous multimodal inputs. On the other hand, the successful application of attention mechanisms and large language models (LLMs) in various fields has inspired new approaches for DTI prediction. For instance, Dong et al. [23] combined graph convolutional networks and transformer modules to extract molecular substructure and semantic information, employing heterogeneous network aggregation to improve prediction performance. AttentionMGT-DTA [24] advances traditional graph-based feature extraction methods by applying graph transformer to the vector representations of drug molecular graphs and protein pocket graphs, thereby achieving more accurate feature modeling. However, these models typically restrict the attention mechanism to feature extraction and fail to extend it to the deeper exploration of relationships between modalities. Moreover, KG_MTL [25] and KGE_UNIT [26], from a multi-task learning perspective, integrate multi-source information from various prediction tasks (e.g., compound-protein interactions, drug-drug interactions), aiding DTI prediction. Overall, these multi-modal DTI prediction methods neglect the interactions between modalities. Their fusion strategies still rely on simple integration methods, such as feature vector concatenation, which merely appends features from different modalities without considering their interdependencies. Shallow self-attention fusion can capture correlations between different features to a certain extent. Nevertheless, it remains constrained by the shallow network architecture, hindering the effective capture of deep interactions between modalities and global information.

We observe that existing models do not fully exploit the multi-relational patterns of drug-target pairs in large-scale KG and the complementary associations between different modalities. These limitations have motivated the development of a novel multimodal learning model, called KG-MACNF. It effectively integrates multi-source relationships in the KG with features derived from other modalities, thereby improving prediction accuracy. We employ RotatE to learn entity representations that capture multiple relations from large-scale KG. Additionally, we utilize an enhanced Graph Attention Network (GAT) and CNN to capture molecular-level features. Finally, the two modality-specific features mentioned above are input into our innovative modality-level fusion network for DTI prediction, which integrates multi-head cross-modal attention and a KAN-driven feedforward neural network. In summary, the contributions of this work are as follows:

i We extract and utilize the rich semantic information from large-scale biological KG and effectively integrate multi-relational features into the representations of biological entities.
ii We design a drug structure representation network, PoolGAT, which integrates GATv2 and significantly enhances prediction performance.
iii We combine cross-modal attention mechanism with a KDFN-based nonlinear mapping approach to develop an innovative fusion framework that enables dynamic interactions across modalities.

## Methods

**Fig 1:**
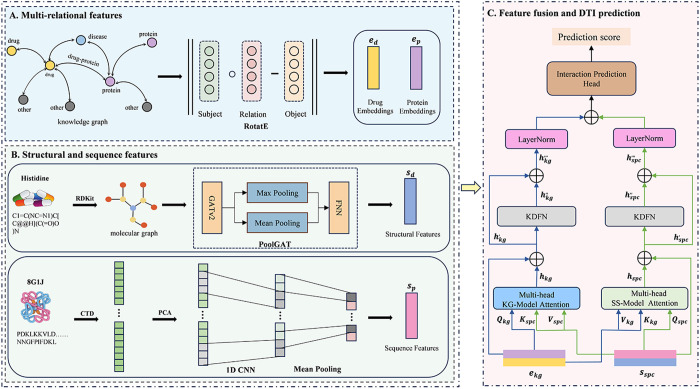
The schematic workflow of KG-MACNF. **(A)** Extraction of multi-relational features of drug and protein entities from the KG. **(B)** Learning feature representations from drug structures and protein sequences. **(C)** Integration of the two modalities and DTI prediction.

Our proposed KG-MACNF (Knowledge Graph-enhanced Modality Attention Cross-Nonlinear Fusion) model resolves the dual limitations of inadequate multi-relational contextual dependency representation and oversimplified multimodal fusion mechanisms prevalent in existing methodologies. KG-MACNF is not only designed to extract global semantic information and structural sequence features for drugs and proteins, but also to integrate these through modality-level attention for DTI prediction. Therefore, this framework consists of three main components, as illustrated in Fig 1.

### Multi-relational features

To resolve the persistent difficulty in distilling complex relational patterns from broad biological contexts into node-level representations, our framework leverages KG as structured semantic descriptors for drug-target characterization. Simultaneously, RotatE [27] is applied to learn the embeddings of entities and relations, as shown in Fig 1A. Specifically, this study represents biomedical concepts (i.e., drugs, proteins, diseases) as entity nodes in the KG, with interactions/associations between nodes as edges. Each relationship in the KG is stored as a triple. For example, a triplet is recorded as <DB15035, interaction, P04626 > , indicating an interaction between drug DB15035 and protein P04626.

To implicitly encode indirect connections between drugs and target proteins into entity representations through multi-hop paths, we adopt the core idea of RotatE. This approach embeds entities and relations into a unified complex space. High-dimensional rotations are employed to capture the multi-level relationships along the connection paths between entities. The embeddings of the head and tail entities in the complex space are initialized as $e_{h}$ and $e_{t}$, respectively, where both $e_{h}$ and $e_{t}$ lie in the complex vector space $C^{k}$. The entity relationships are denoted as $e_{r}\;\in \;C^{k}$. For each record in the triple, we aim to map the relationship as an element-wise rotation from the head entity to the tail entity:


$$e_{t}\;=e_{h}\;\circ \;e_{r}$$ (1)

where ◦ denotes the element-wise product. To verify the operational effectiveness for capturing multi-level semantics, we design the following distance function $d_{r}(e_{h},e_{t})$. The function measures the alignment between the head entity embedding $(e_{h})$, transformed by the rotation operation of the relation embedding $(e_{r})$, and the tail entity embedding $(e_{t})$ in the complex vector space:


$$d_{r}(e_{h},e_{t})=∥\;\begin{array}{c} e_{h}\circ e_{r}-e_{t} \end{array}\;∥$$ (2)

where ∥ ⋅ ∥ denotes the $L_{2}$ paradigm for computing the Euclidean distance between complex vectors. A smaller distance indicates a stronger semantic link between the head and tail entities. By training this scoring function, we obtain the representations of all entities. In practice, we derive only the embeddings corresponding to drugs and proteins, denoted as $e_{d}$ and $e_{p}$, respectively.

### Structural and sequence features

In addition to the above global knowledge, the microscopic features of drugs and proteins also play a crucial role in DTI prediction. Through these features, we are able to reveal the potential mechanisms of interaction between drugs and proteins at a molecular level. For example, the molecular structure of a drug determines its compatibility with the active site of a protein, yet specific patterns within amino acid sequences influence drug binding affinity. Such fine-grained information is essential for a comprehensive understanding of drug-target relationships.

#### Drug structure representation.

**Fig 2:**
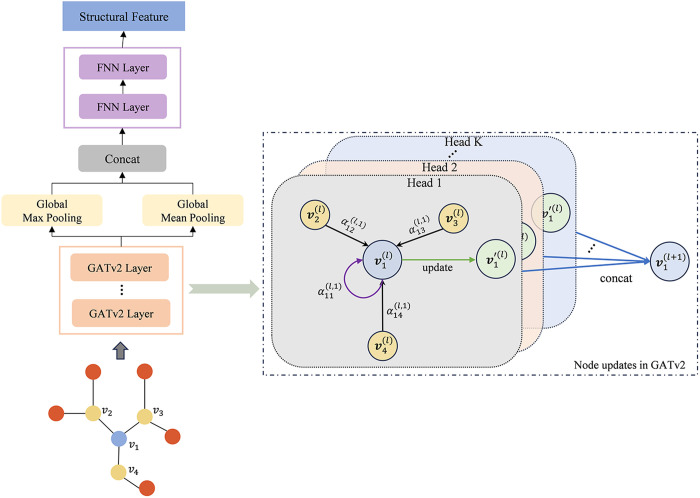
PoolGAT network architecture.

The structure of a drug is a key determinant of its biological activity and mechanism of action. To capture these structural characteristics, we design a molecular graph-specific feature extraction process, as depicted in Fig 1B. The SMILES (Simplified Molecular Input Line Entry System) is converted into graphs using RDKit [28]. At the same time, the properties listed in Table 1 are used as features of the nodes in the molecular graph. These are encoded through one-hot encoding to form the node feature set $D=\left\{v_{1},v_{2},\ldots ,v_{\left|D\right|}\right\}$, where |D| represents the total number of nodes. To extract high-quality features from graphical data, we employ PoolGAT, a network designed for effective feature learning, with its detailed structure presented in Fig 2.

**Table 1:** Node features (atom).

Property	Dimension	Value
The atom symbol	44	[‘C’, ‘N’, ‘O’, ‘S’, ‘F’, ‘Si’, ‘P’, ‘Cl’, ‘Br’, ‘Mg’, ‘Na’, ‘Ca’, ‘Fe’, ‘As’, ‘Al’, ‘I’, ‘B’, ‘V’, ‘K’, ‘Tl’, ‘Yb’, ‘Sb’, ‘Sn’, ‘Ag’, ‘Pd’, ‘Co’, ‘Se’, ‘Ti’, ‘Zn’, ‘H’, ‘Li’, ‘Ge’, ‘Cu’, ‘Au’, ‘Ni’, ‘Cd’, ‘In’, ‘Mn’, ‘Zr’, ‘Cr’, ‘Pt’, ‘Hg’, ‘Pb’, ‘other’]
The number of adjacent atoms	11	[0, 1, 2, 3, 4, 5, 6, 7, 8, 9, 10]
The number of adjacent hydrogens	11	[0, 1, 2, 3, 4, 5, 6, 7, 8, 9, 10]
The implicit value of the atom	11	[0, 1, 2, 3, 4, 5, 6, 7, 8, 9, 10]
Whether the atom is aromatic	1	[0,1]

According to the complementarity of local and global features within the molecule, PoolGAT updates the features of each node in the molecular graph progressively through multi-layer GATv2 [29]. In this process, the self-attention mechanism is employed to adaptively compute the weighted aggregation of neighborhood features, integrating this information into the target node, while self-loops are introduced to retain the node’s own features. Additionally, to enhance the stability of learning in the self-attention mechanism, we incorporate a multi-head attention mechanism. Multiple attention heads aim to capture node features from different perspectives, and their outputs are concatenated to generate the hidden layer feature representation of each node:


$$v_{i}^{\left(l+1\right)}=\sigma {(∥}_{k=1}^{K}(\sum_{j\in {𝒩}_{i}}\;\alpha_{ij}^{\left(l,k\right)}W^{\left(l,k\right)}v_{j}^{\left(l\right)}))\mathrm{\backslash ]}$$ (3)

where ${\text{N}}_{i}$ is the set of neighbors of vertex *i*, and *K* represents the number of attention heads. $W^{\left(l,k\right)}$ is the learnable weight matrix of the *k*-th attention head at the *l*-th layer. ∥ denotes the concatenation operation, and *σ* represents the activation function. The attention coefficient $\alpha_{ij}^{\left(l\text{,}k\right)}$ is calculated based on the feature simi*l*arity between nodes *i* and *j*:


$$\alpha_{ij}^{\left(l\text{,}k\right)}=\frac{a^{T}LeakyReLU\left(W^{\left(l,k\right)}\left[v_{i}^{\left(l\right)}∥v_{j}^{\left(l\right)}\right]\right)}{\sum_{m\in {\text{N}}_{i}}\;\text{exp}\left(a^{\text{T}}LeakyReLU\left(W^{\left(l,k\right)}\left[v_{i}^{\left(l\right)}∥v_{m}^{\left(l\right)}\right]\right)\right)}$$ (4)

where ***a*** is a learnable weight vector, and *LeakyReLU* is the activation function. After *L* rounds of message aggregation, the final feature representation of each vertex is obtained as $D^{\left(L\right)}\left\{v_{1}^{\left(L\right)},v_{2}^{\left(L\right)},\ldots ,v_{\left|D\right|}^{\left(L\right)}\right\}$. To obtain the global features of the molecular structure, a dual-channel pooling strategy is applied to integrate the local features of the nodes. The channel of max pooling facilitates the highlighting of key nodes in the molecular structure and the retention of critical features associated with bioactivity. In contrast, a balanced representation of the molecule is achieved by uniformly integrating the contributions of all nodes through the channel of average pooling. The concatenated results of the two pooling operations are fed into a fully connected network (FNN) for feature mapping, resulting in a fixed-dimensional representation of the drug molecule structure. The final feature $s_{d}$ of each drug molecule structure is obtained as shown in formula (5).


$$s_{d}=FNN(concat[\underset{v_{i}\in D^{\left(L\right)}}{maxpool}\;\left(v_{i}\right),\underset{v_{i}\in D^{\left(L\right)}}{meanpool}\;\left(v_{i}\right)])$$ (5)

where *maxpool* denotes taking the maximum value element-wise across each feature dimension, while *meanpool* calculates the mean value dimension-wise. Both operations produce outputs with dimensions consistent with the node feature size. *concat* represents the concatenation operation, *FNN* refers to the fully connected layer.

#### Protein sequence representation.

Protein characteristics, such as sequence, secondary structure, hydrophobicity, and charge distribution, provide critical insights into potential drug binding sites. As illustrated in Fig 1B, the protein representation process employs Composition-Transition-Distribution (CTD) descriptors to convert amino acid sequences of 20 protein symbols into high-dimensional feature vectors [22]. It captures features related to specific structural or physicochemical properties through the amino acid distribution and transition patterns within the sequence. 13 types of physicochemical properties have been utilized in previous studies to calculate these features, including hydrophobicity, normalized lorry volume, polarity, polarizability and solvent accessibility. To address the high dimensionality of CTD features, principal component analysis (PCA) [30] is applied to reduce complexity and redundancy while the essential information is retained. A network comprising a convolutional layer followed by a pooling layer is used to extract latent local patterns from the protein sequence. The forward propagation in the convolutional layer is described by the following equation:


$$s_{p}=Pooling(\sigma (W_{c}*p_{PCA}+b_{c}))$$ (6)

where $\text{\textbackslash{} }W_{c}$ denotes the filter, * represents the convolution operation, and $b_{c}$ is the bias vector. $p_{PCA}$ is the input which is processed by CTD and PCA, and *Pooling* refers to the average pooling. The final output of the protein representation is denoted as $s_{p}$.

### Feature fusion and DTI prediction

**Fig 3:**
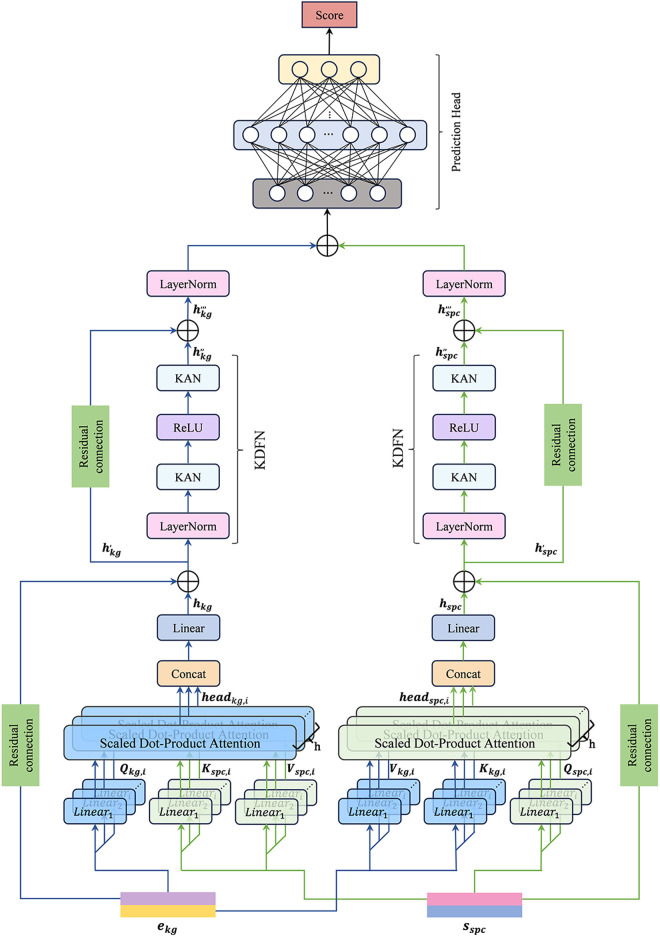
Detailed structure of feature fusion and DTI prediction modules.

Feature fusion is pivotal in enhancing prediction accuracy for multimodal DTI task. While previous studies primarily emphasized integrating additional modalities, they frequently overlooked the heterogeneity and limitations of fusion strategies in multimodal interaction. Simplistic feature combinations from different modalities frequently introduce conflicts and noise, limiting the model’s predictive ability. To address these challenges, we propose a novel multimodal feature fusion network, as illustrated in Fig 3. This network integrates features from Modules A and B (Fig 1) through a cross-modal attention mechanism and a KDFN-based nonlinear mapping approach enabling precise prediction of DTI.

#### Modality-level feature fusion.

This network is designed with dual modality-specific processing flows. The features $e_{d}$ and $e_{p}$ are concatenated to form $e_{kg}$, while $s_{d}$ and $s_{p}$ are combined to produce $s_{spc}$ which serve as the network inputs. To address the issue of excessive focus on a particular relational pattern during modality interaction, we introduce a multi-head attention mechanism [31] and design two multi-head modality attention layers: the Multi-Head Knowledge Graph Attention (MKGA) layer and the Multi-Head Structural-Sequence Attention (MSSA) layer. These map the modality interaction into multiple subspaces, with each attention head focusing on distinct correlation patterns.

The two attention layers share similar structures but differ in the entities used as queries, keys, and values to exploit complementary modality interactions effectively. In the MKGA layer, the global semantic relationships from the KG modality as queries guide the weighted aggregation of structural sequence features. Therefore, the query $Q_{kg}$ is generated from the KG representation $e_{kg}$, while the key $K_{spc}$ and value $V_{spc}$ are derived from the structural sequence feature $s_{spc}$. Conversely, in the MSSA layer, the structural sequence feature serves as queries to refine the embeddings of knowledge-based entities. The $Q_{spc}$ is obtained from $s_{spc}$, whereas the $K_{kg}$and $V_{kg}$ are provided by $e_{kg}$. The formal expression of this process is provided below:


$$\left\{ \begin{array}{c} Q_{kg,i},K_{kg,i},V_{kg,i}={Linear}_{i}(e_{kg})\\ Q_{spc,i},K_{spc,i},V_{spc,i}={Linear}_{i}(s_{spc}) \end{array}\right.\;$$ (7)

$$\left\{ \begin{array}{c} {head}_{kg,i}=Softmax\left(\frac{Q_{kg,i}K_{spc,i}^{T}}{\sqrt{d_{k}}}\right).V_{spc,i}\\ {head}_{spc,i}=Softmax\left(\frac{Q_{spc,i}K_{kg,i}^{T}}{\sqrt{d_{k}}}\right).V_{kg,i} \end{array}\right.\;$$ (8)

$$\left\{ \begin{array}{c} h_{kg}=Linear(concat\left({head}_{kg,1},{head}_{kg,2},\ldots ,{head}_{kg,N}\right))\\ h_{spc}=Linear(concat\left({head}_{spc,1},{head}_{spc,2},\ldots ,{head}_{spc,N}\right)) \end{array}\right.\;$$ (9)

where ${Linear}_{i}$ represents the independent linear transformation for each head, while $Q_{\sim ,i},K_{\sim ,i},V_{\sim ,i}$ separately denote the query, key, and value, which are the linear projections of different modalities.${\;d}_{k}$ is the scaling factor, and *Softmax* refers to the normalized exponential function. ${head}_{kg,i}$ and ${head}_{spc,i}$ represent the outputs of the *i*-th head for the two modalities, and *N* is the number of attention heads.

The cross-attention mechanisms may cause an over-smoothing representation of features. To prevent the loss of original feature details from both modalities, the outputs of MKGA and MSSA ($h_{kg}\text{, }h_{spc}$) are added to their corresponding original modality features ($e_{kg}\text{, }s_{spc}$) through a skip connections, resulting in the final cross-fused outputs ($h_{kg}^{\text{,}}\text{ , }h_{spc}^{\text{,}}$).

#### KDFN and prediction head.

Cross-attention primarily focuses on interactions between different modalities, capturing dependencies through weight distribution. Nonetheless, its output may remain a local linear mapping, possibly constraining its capacity for executing intricate nonlinear transformations. Therefore, we design a Kolmogorov–Arnold Network (KAN)-driven feedforward neural network (KDFN) in each processing flow, as detailed in Fig 3. To ensure consistent distribution of the input features, layer normalization is applied before the KAN layer:


$$\left\{ \begin{array}{c} x_{kg}=LN(h_{kg}^{,})\\ x_{spc}=LN(h_{spc}^{,}) \end{array}\right.\;$$ (10)

where *LN* denotes layer normalization, the components of $x_{kg}$ and $x_{spc}$ are given by $x_{kg}={[x}_{kg,1},x_{kg,2},\ldots x_{kg,n}$] and $x_{spc}=[x_{spc,1},x_{spc,2},\ldots x_{spc,n}$], *n* is the dimension of the vector.

In DTI prediction, the essential interactions between molecular structures and protein sequences frequently display complex, high-order nonlinear characteristics. Conventional feedforward networks rely on linear projections followed by static activation functions, resulting in constraining their ability to represent intricate nonlinear patterns. To address this limitation, we adopt KAN [32] as a replacement for standard linear layers in the KDFN module. KAN explicitly learns B-spline basis functions for each input feature, enabling data-dependent nonlinear transformations. Instead of relying on a pre-defined function shape, the learned B-spline curves flexibly adapt to the data distribution, capturing subtle nonlinear variations. These basis functions are defined over adaptive grids and can approximate a wide range of higher-order functional relationships without relying on deep layer stacking, enhancing the expressiveness and interpretability of feature mapping. The specific computation process is as follows:


$$\left\{ \begin{array}{c} x_{kg}^{\prime }=KAN\left(x_{kg}\right)=\sum_{q=1}^{2n+1}\emptyset_{q}(\sum_{p=1}^{n}\varphi_{q,p}(x_{kg,p}))\\ x_{spc}^{\prime }=KAN\left(x_{spc}\right)=\sum_{q=1}^{2n+1}\emptyset_{q}(\sum_{p=1}^{n}\varphi_{q,p}(x_{spc,p})) \end{array}\right.\;$$ (11)

$$\left\{ \begin{array}{c} h_{kg}^{,,}=KAN\left(ReLU\left(x_{kg}^{\prime }\right)\right)\\ h_{spc}^{,,}=KAN(ReLU\left(x_{spc}^{\prime }\right)) \end{array}\right.\;$$ (12)

where $\;x_{kg,p}$ and $x_{spc,p}$ represent the components of the feature vectors from the two modalities, with each ∅ being a real-valued function and *ReLU* serving as the activation function. Each $\varphi_{q,p}$ is a unique activation function in KAN, defined as follows:


$$\varphi (x)=\;w_{b}b(x)+w_{s}\;Bspline(x)$$ (13)

$$b(x)=\;\frac{x}{1+e^{-x}}$$ (14)

where *x* represents the activation value input to the neuron, while $w_{b}$ and $w_{s}$ are learnable weights that regulate the contribution of each component. *Bspline* denotes the B-spline basis functions. Similarly, we append a residual structure after the KDFN to prevent gradient vanishing. Thus, the updated representations $h_{kg}^{,,,}$ and $h_{spc}^{,,,}$ are obtained by formula (15):


$$\left\{ \begin{array}{c} {h_{kg}^{,,,}=\;h}_{kg}^{,,}+h_{kg}^{,}\\ {h_{spc}^{,,,}=\;h}_{spc}^{,,}+h_{spc}^{,} \end{array}\right.\;$$ (15)

To obtain the final prediction, the representations $h_{kg}^{\text{,,,}}$ and $h_{spc}^{\text{,,,}}$ are first normalized using layer normalization and then concatenated. Subsequently, the result is passed through the prediction head, which consists of fully connected layers, a batch normalization layer and activation layers, to compute the predicted scores:


$$y=\partial_{2}(W_{2}\left(BN(\partial_{1}(W_{1}\left(Flatten\left(LN\left(h_{kg}^{,,,}\right)∥LN(h_{spc}^{,,,})\right)\right)+b_{1}))\right)+b_{2})$$ (16)

where *Flatten* denotes the flattening operation, while $W_{i}$, $b_{i}$, and $\partial_{i}$ represent the weight matrix, bias vector, and activation function for the *i*-th layer, respectively. *BN* refers to the batch normalization.

### Training strategy

Since the KG contains only positive triples, random negative sample noise is introduced during the training phase to enhance embedding quality. We employ a self-adversarial negative sampling method to optimize the knowledge embeddings:


$$\begin{array}{c} {\text{L}}_{KG}=-\text{log}\;\rho (\gamma -d_{r}(e_{h}\text{,}e_{t}))-\sum_{i=\text{1}}^{n}\;p(h_{i}^{\prime }\text{,}r\text{,}t_{i}^{\prime })\text{log }\rho (d_{r}(e_{\left(h\text{,}i\right)}^{\prime }\text{,}e_{\left(t\text{,}i\right)}^{\prime })-\gamma ) \end{array}$$ (17)

where ρ is the optimization function, γ denotes a fixed margin, and $\left(h_{i}^{\prime }\text{,}{r\text{,}t}_{i}^{\prime }\right)$ represents the *i*-th negative triplet. $p(h_{i}^{\prime }\text{,}r\text{,}t_{i}^{\prime })$ is used to transform the score of the negative sample into a probability:


$$\begin{array}{c} p(h_{j}^{\prime }\text{,}r\text{,}t_{j}^{\prime }|\{(h_{i}\text{,}r_{i}\text{,}t_{i})\})=\frac{exp\left(\alpha d_{r}\left(e_{\left(h\text{,}j\right)}^{\prime }\text{,}e_{\left(t\text{,}j\right)}^{\prime }\right)\right)}{\sum \text{exp}\left(\alpha d_{r}\left(e_{\left(h\text{,}i\right)}^{\prime }\text{,}e_{\left(t\text{,}i\right)}^{\prime }\right)\right)} \end{array}$$ (18)

where α is the sampling temperature. Additionally, for the DTI prediction task, our optimization objective is to minimize the following cross-entropy loss:


$${\text{L}}_{dti}=-\frac{\text{1}}{N}\sum_{i=\text{1}}^{N}\;\left[y_{i}\cdot \text{log}({\hat{y}}_{i})+(\text{1}-y_{i})\cdot \text{log}(\text{1}-{\hat{y}}_{i})\right]+\lambda \cdot \sum_{m=\text{1}}^{M}\;||W_{m}|{|}_{\text{2}}^{\text{2}}\mathrm{\backslash ]}$$ (19)

where *N* is the number of samples, ${\hat{y}}_{i}$ is the predicted output and $y_{i}$ is the true label (0 or 1). λ represents the weight decay, *M* is the number of weight matrices and $W_{m}$ denotes the *m*-th weight matrix.

## Results

### Dataset

In this study, we evaluate our proposed model by using two benchmark datasets: Yamanishi_08’s [33] and BioKG [34]. These two datasets were selected to reflect complementary characteristics in terms of data scale and biological complexity. Yamanishi_08’s serves as a focused, small-scale benchmark, while BioKG provides a large-scale, heterogeneous biomedical KG. This design facilitates a thorough assessment of the model’s generalization ability across diverse and realistic biological scenarios.

#### Yamanishi_08’s.

The dataset consists of four sub-datasets collected from KEGG, BRENDA, SuperTarget and DrugBank, namely enzymes (E), ion channels (IC), G-protein coupled receptors (GPCR), and nuclear receptors (NR). We construct a knowledge graph that contains 25,487 unique nodes and 95,579 edges, based on these four sub-datasets.

#### BioKG.

The dataset integrates data from 14 biomedical databases, including entities related to drugs, proteins, genes, diseases, and more. In this experiment, we randomly select half of the drug and protein entities while preserving their relationships, resulting in a sub-knowledge graph containing 49,615 unique nodes and 513,160 edges.

Due to the absence of negative samples in the original dataset, we treated known DTI pairs as positive samples and randomly selected non-existing DTI pairs to generate negative samples. To accurately represent the inherent sparsity of verified drug–target interactions in actual biological networks, we constructed both balanced (1:1) and imbalanced (1:10) datasets. The 1:10 setting, in particular, aligns with commonly adopted practices in prior research and introduces sufficient challenge while ensuring training stability.

### Baselines and evaluation protocols

We selected a diverse set of baseline methods spanning machine learning, deep learning, knowledge graph embedding, and hybrid models to comprehensively evaluate our method and analyze performance trends across different modeling paradigms.

- Random Forest (RF) and Support Vector Machine (SVM) utilize Extended Connectivity Fingerprints (ECFP) of drug compounds and the PSC features of protein descriptors as input.
- DeepDTI takes SMILES and FASTA sequences as input and applies a neural network based on Restricted Boltzmann Machine to extract ECFP and PSC features for DTI prediction [35].
- GNN-CPI employs GNN to encode drug molecular graphs and CNN to extract amino acid sequence features of proteins for the CPI task [10].
- TriModel is a heterogeneous data-driven approach based on the use of KG embeddings to obtain representations of entities in heterogeneous networks for DTI task [16].
- KGE_NFM combines multi-source information by integrating KG embeddings with molecular fingerprints and protein CTD descriptors as input, using Neural Factorization Machine (NFM) to capture higher-order interactions for DTI prediction [17].
- TransE, ConvE, ComplEx, DistMult, CrossE, RotatE, and PairRE are various knowledge graph embedding (KGE) methods, directly applied to DTI task [27,36–41].

All experimental parameters for the baselines follow the original settings. we employ 10-fold cross-validation (10-CV) to compare the performance of proposed method with others. Area under the receiver operating characteristic curve (AUC) and area under the precision-recall curve (AUPR) are adopted as evaluation metrics. Specifically, we evaluate under the following three scenarios:

1. warm start: The training and test sets share common drugs and proteins.
2. cold start for drug: The test set contains only drugs that are unseen in the training set, while all proteins are shared in both sets.
3. cold start for protein: The test set contains only proteins that are unseen in the training set, while all drugs are shared in both sets.

### Hyperparameter settings

All experiments were conducted in a Python 3.8 environment with PyTorch 1.13.1, using an NVIDIA RTX 3060 GPU with 12GB of memory and running on a Linux operating system. A summary of all the hyperparameters is provided in Table 2. To ensure reproducibility and accuracy, the following hyperparameter settings were determined after multiple rounds of experimentation.

**Table 2:** The hyperparameters of KG-MACNF.

Parameters	Value
Entity Embedding Dimension	512
Epochs	150
Learning Rate	0.001
Batch Size	512
Dropout	0.1
Weight Decay	0.001
Number of GATv2 Layers	2
Optimizer	Adam
Multi-Head attention	8

Notably, four important parameters—Entity Embedding Dimension, Number of GATv2 Layers, Learning Rate and Weight Decay—are analyzed in detail as follows:

(1) **Impact of Entity Embedding Dimension**

**Fig 4:**
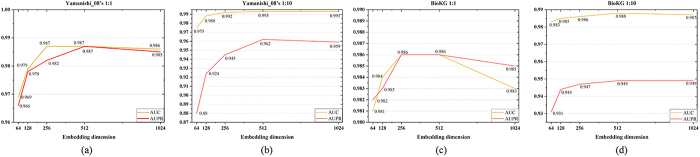
Impact of different KG embedding dimensions on KG-MACNF.

We evaluate the influence of dimension of entity embedding on KG-MACNF by varying it from 64 to 1024. Fig 4 illustrate that the optimal performance is achieved with a 512-dimensional embedding on both the Yamanishi_08’s and BioKG datasets. As the dimension increases from 64 to 512, the model’s performance improves gradually. However, when the dimension reaches 1024, a slight decline is observed. It implies that a 512-dimensional embedding provides sufficient representational capacity while avoiding the introduction of redundant features.

(2) **Impact of the Number of GATv2 Layers**

**Fig 5:**
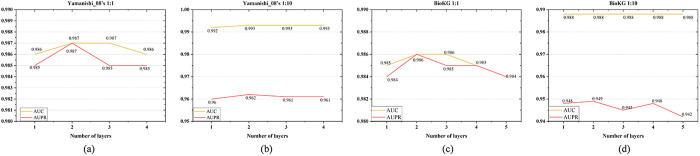
Impact of different GATv2 layer numbers on KG-MACNF.

As shown in Fig 5, we vary the number of GATv2 layers from 1 to 4. The results indicate that increasing the layers from 1 to 2 results in slight improvements for AUC and AUPR. Feature extraction can be improved by appropriately deepening the GATv2 layers. However, when the number of layers is further increased to 3 or more, some metrics (i.e., AUC and AUPR on the balanced dataset, and AUPR on the imbalanced dataset) slightly decrease, suggesting that excessive layers may lead to feature smoothing, reducing model’s generalization ability. Given the observed trends, a two-layer GATv2 architecture demonstrates optimal performance for this task.

(3) **Impact of the Learning Rate**

**Fig 6:**
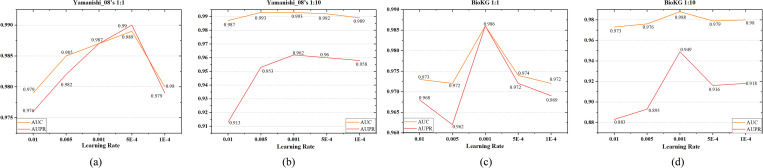
Impact of different learning rates on KG-MACNF.

To assess the impact of the learning rate, we test five widely utilized values during hyperparameter optimization: {0.01, 0.005, 0.001, 0.0005, 0.0001}, covering a wide range of training dynamics. As illustrated in Fig 6, the model performance remains relatively stable across these settings, with the difference between the highest and lowest values on both metrics not exceeding 0.07. In most cases, particularly on BioKG and Yamanishi_08’s (1:10), the best results are achieved when the learning rate is set to 0.001. Although slightly higher scores are observed on Yamanishi_08’s (1:1) at other values, the overall trend supports 0.001 as a balanced and effective choice. These results further indicate that the model is robust to reasonable variations in the learning rate.

(4) **Impact of Weight Decay**

**Fig 7:**
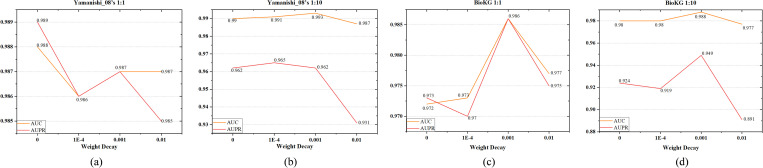
Impact of different weight decay values on KG-MACNF.

We evaluate the effect of weight decay by selecting four values in increasing order. As shown in Fig 7, under balanced settings, performance first decreases, then improves, and finally declines again as the regularization strength increases. BioKG attains optimal performance with a weight decay of 0.001, while Yamanishi_08’s (1:1) excels in the absence of weight decay, as the model avoids overfitting and unnecessary regularization does not constrain its ability to discern significant patterns. In imbalanced scenarios, performance remains relatively stable when weight decay is set between 0 and 0.001, but drops significantly as the regularization becomes too strong, indicating underfitting. Overall, setting the weight decay to 0.001 enhances generalization without hindering convergence and yields consistently satisfactory results across datasets.

### Performance evaluation under the warm start scenario

To ensure a fair evaluation of each model, we apply 10-CV to compare KG-MACNF with 13 baseline methods on the two benchmark datasets mentioned earlier. In this process, each dataset is randomly divided into 10 subsets. In each iteration, 9 subsets are used for the training set, and the remaining subset serves as the test set. The final performance metric is the average of the 10 iterations, as shown in Table 3. It can be observed that regardless of the positive-to-negative sample ratio, KG-MACNF consistently outperforms all baselines on both datasets. In the balanced case of the Yamanishi_08’s dataset, the AUC improves by a minimum of 2.2%, and the AUPR shows an increase of by at least 3.5%. In the imbalanced case, the AUC achieves an improvement of at least 0.6%, and the AUPR increases at least 3.8%. On the BioKG dataset, the improvements are 0.8% and 0.6% in the 1:1 ratio case and 0.3%, 2.2% in the 1:10 ratio case, respectively. Notably, the AUPR improvement is most significant in the imbalanced case. It suggests that our model can effectively integrate multimodal features and still learn valuable information, even with sparse positive samples. Compared to end-to-end models which only use drug and protein-specific features (e.g., DeepDTI and GNN_CPI), KG-MACNF leverages additional biological relationships from knowledge graphs, leading to superior performance. Furthermore, under imbalanced dataset conditions, KGE_NFM and various KGE methods show a decrease in AUPR, with the former experiencing a smaller reduction. This further supports the robustness of multimodal fusion methods in handling data imbalance.

**Table 3:** Performance on Yamanishi_08’s and BioKG dataset in the scenario of the warm start.

	Yamanishi_08’s				BioKG			
Pos/Neg Ratio	1:1		1:10		1:1		1:10	
Metrics	AUC	AUPR	AUC	AUPR	AUC	AUPR	AUC	AUPR
KG-MACNF	0.987	0.987	0.993	0.962	0.986	0.986	0.988	0.949
SVM	0.601	0.715	0.728	0.557	0.714	0.785	0.796	0.598
RF	0.873	0.872	0.923	0.687	0.978	0.98	0.98	0.927
DeepDTI	0.863	0.826	0.983	0.924	0.976	0.974	0.981	0.912
GNN_CPI	0.847	0.812	0.975	0.884	0.958	0.96	0.961	0.847
TriModel	0.952	0.946	0.987	0.892	0.953	0.954	0.982	0.875
KGE_NFM	0.965	0.952	0.984	0.913	0.959	0.948	0.985	0.892
TransE	0.824	0.799	0.894	0.568	0.885	0.899	0.936	0.793
ConvE	0.856	0.841	0.92	0.675	0.903	0.909	0.935	0.816
ComplEx	0.828	0.795	0.912	0.676	0.887	0.902	0.938	0.822
Distmult	0.917	0.916	0.934	0.706	0.925	0.927	0.928	0.79
CrossE	0.921	0.914	0.939	0.766	0.945	0.951	0.96	0.863
RotatE	0.946	0.95	0.952	0.864	0.948	0.964	0.969	0.875
PairRE	0.927	0.932	0.941	0.828	0.945	0.949	0.949	0.835

### Comparative analysis of different KGE methods

**Fig 8:**
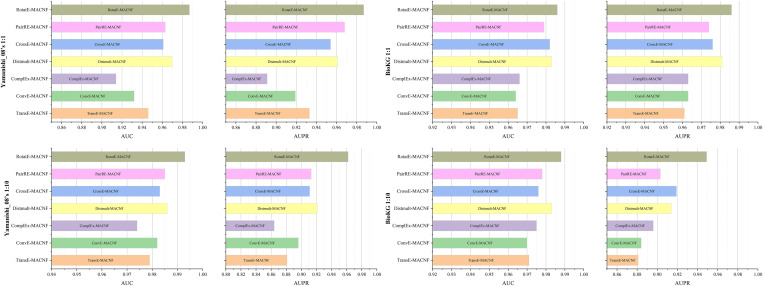
Prediction performance of KG-MACNF with different KGE models.

To assess the impact of different KGE methods on the DTI prediction performance of our model, we replace RotatE in the original model with other embeddings such as TransE and ComplEx, and conduct comparative experiments on the Yamanishi_08’s and BioKG datasets. As shown in Fig 8, RotatE consistently outperforms other embedding methods across all evaluation metrics. This suggests that RotatE excels at capturing the global multi-relational context of drug-target interactions within biological networks, highlighting its strong capability in deeply exploring the semantics of KG. Furthermore, compared to the models that use KGE alone (as presented in Table 3), integrating our proposed module leads to varying degrees of performance improvement across all embedding methods, with TransE and ComplEx showing the most significant improvements. Specifically, on the Yamanishi_08’s dataset, the AUC and AUPR of TransE improve by an average of 10.3% and 22.3%, and ComplEx achieves gains of 7.4% in AUC and 14.2% in AUPR. Similarly, TransE shows an average improvement of 5.7% in AUC and 7.5% in AUPR, and the AUC and AUPR of ComplEx demonstrates gains of 5.8% and 6.7%, respectively, on the BioKG dataset. These demonstrate that our model not only efficiently utilizes high-quality embedding methods but also achieves substantial improvements with relatively lower-quality embeddings, further highlighting its adaptability and robustness in handling different KGE techniques.

### Performance evaluation under the cold start scenario

We further evaluate the performance of KG-MACNF in both drug and protein cold start scenarios. Similarly, 10-CV is applied for all experiments in this section.

#### Prediction results in the scenario of cold start for drugs.

As shown in Table 4, in the scenario of the cold start for drugs, KG-MACNF outperforms all methods on the Yamanishi_08’s dataset (AUC = 0.871, AUPR = 0.874), while RF attains the highest AUPR on the BioKG dataset (AUC = 0.917, AUPR = 0.932). However, a general decline in performance is observed across all models under this setting. We attribute this degradation primarily to the limited background information available for new drugs. The absence of prior interaction records or structural features in the training set hinders the model’s ability to generalize to previously unseen compounds. Moreover, in this setting, the model’s reliance on KGE may become a limiting factor, as KGE approaches heavily depend on pre-existing connections in the graph, which are frequently remain sparse or altogether missing for new drugs.

**Table 4:** DTI prediction in drug cold start on Yamanishi_08’s and BioKG (Balanced).

	Yamanishi_08’s		BioKG	
Metrics	AUC	AUPR	AUC	AUPR
KG-MACNF	0.871	0.874	0.925	0.924
SVM	0.586	0.67	0.683	0.767
RF	0.787	0.803	0.917	0.932
DeepDTI	0.694	0.743	0.869	0.874
GNN_CPI	0.773	0.787	0.855	0.867
TriModel	0.748	0.755	0.858	0.853
KGE_NFM	0.806	0.794	0.899	0.906
TransE	0.713	0.694	0.788	0.815
ConvE	0.7	0.689	0.782	0.814
ComplEx	0.707	0.696	0.797	0.829
Distmult	0.669	0.643	0.8	0.822
CrossE	0.751	0.744	0.809	0.833
RotatE	0.774	0.75	0.85	0.865
PairRE	0.738	0.735	0.831	0.85

Overall, models incorporating drug structural representations generally perform better than those relying solely on KGE methods. This suggests that when drug background information is limited, structural features effectively compensate for the shortcomings of KGE. The performance of KG-MACNF in cold start settings further highlights the importance of integrating richer drug representations to enhance the model’s generalization capability in real-world biomedical applications.

#### Prediction results in the scenario of cold start for proteins.

In the scenario of the cold start for proteins, as presented in Table 5, KGE_NFM achieves the best performance on the Yamanishi_08’s dataset (AUC = 0.901, AUPR = 0.877). On the BioKG dataset, KG-MACNF demonstrates the best performance (AUC = 0.81, AUPR = 0.831), outperforming all baseline methods. All models exhibit a significant decline in performance compared to the drug cold start scenario, especially those that rely heavily on protein features for prediction (e.g., RF). This indicates that, without prior training knowledge, the generalization ability of models to novel proteins is more limited. Compared to molecules with relatively well-defined and regular structures, proteins typically exhibit higher-dimensional folding patterns, more intricate spatial conformations, and dynamically varying functional domains. These characteristics make their biological properties difficult to capture using simple 1D or 2D representations. Under such conditions, both sequence-based and graph-based features may inadequately convey critical functional information, limiting the model’s ability to comprehend unseen proteins. In contrast, KG-MACNF and KGE_NFM demonstrate enhanced robustness, indicating that their multimodal fusion strategies help alleviate the shortcomings of individual protein feature representations.

**Table 5:** DTI prediction in protein cold start on Yamanishi_08’s and BioKG (Balanced).

	Yamanishi_08’s		BioKG	
Metrics	AUC	AUPR	AUC	AUPR
KG-MACNF	0.871	0.865	0.81	0.831
SVM	0.53	0.654	0.559	0.659
RF	0.271	0.369	0.287	0.368
DeepDTI	0.472	0.515	0.514	0.505
GNN_CPI	0.506	0.574	0.501	0.515
TriModel	0.797	0.785	0.782	0.774
KGE_NFM	0.901	0.877	0.739	0.782
TransE	0.47	0.467	0.671	0.678
ConvE	0.571	0.529	0.636	0.644
ComplEx	0.553	0.566	0.62	0.613
Distmult	0.642	0.64	0.641	0.623
CrossE	0.747	0.721	0.686	0.681
RotatE	0.685	0.629	0.753	0.757
PairRE	0.732	0.723	0.773	0.78

### Ablation study

To investigate the contributions of knowledge embedding, structural sequence features, and the multimodal fusion network to the performance of KG-MACNF, we conduct the ablation study on the BioKG dataset with the following variants:

- **KG-MACNF-HF**: This variant uses only the contextual embedding features of drugs and proteins learned from the KG.
- **KG-MACNF-SF**: This variant uses only the individual features learned from the independent representations of drugs and proteins.
- **KG-MACNF-HS**: This variant uses the integrated features formed by concatenating contextual embeddings and individual features.

As shown in Table 6, while both KG-MACNF-HF and KG-MACNF-SF exhibit some predictive abilities, they consistently underperform compared to other variants. Although KG-MACNF-HS shows improvements over models using only a single modality, it still falls short of outperforming the original model. In particular, in the imbalanced scenario, KG-MACNF-HS shows a more pronounced decline in AUPR, whereas KG-MACNF remains relatively stable. This proves that the combination of the cross-attention mechanism and nonlinear mapping in the original model contributes significantly to the predictive performance.

**Table 6:** Ablation study result.

	BioKG			
Pos/Neg Ratio	1:1		1:10	
Metrics	AUC	AUPR	AUC	AUPR
KG-MACNF-HF	0.948	0.964	0.969	0.875
KG-MACNF-SF	0.95	0.947	0.946	0.824
KG-MACNF-HS	0.973	0.979	0.973	0.928
KG-MACNF	0.986	0.986	0.988	0.949

**Fig 9:**
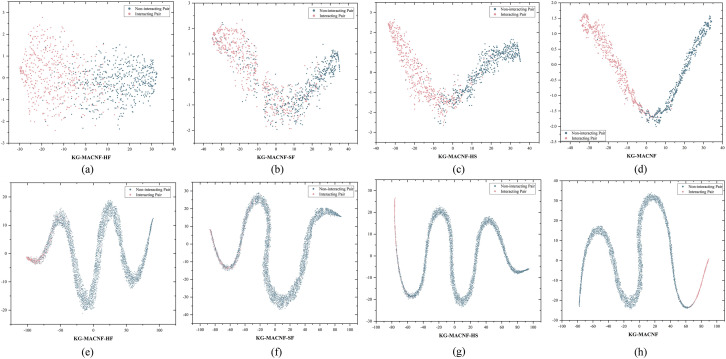
Feature embeddings learned by different ablation variants on the Yamanishi_08’s test set. (a-d) visualization under balanced conditions, (e-h) visualization under imbalanced conditions.

To further visualize the classification performance of each model variant, we apply T-SNE [42] to project the features learned by different ablation models into a two-dimensional space. The results on the Yamanishi_08’s dataset are shown in Fig 9. For instance, under balanced data conditions (a-d), we observe the following: KG-MACNF-HF, although capable of distinguishing some samples, exhibits a relatively scattered feature distribution. KG-MACNF-SF shows a tendency towards clustering, but its classification performance is significantly flawed. In contrast, KG-MACNF-HS, which combines multiple biological features, contains more discriminative information, resulting in both improved clustering and classification performance. Lastly, KG-MACNF, which dynamically focuses on key information during feature fusion through attention mechanisms, effectively distinguishes between different sample categories. The aggregation of samples within the same class is also significantly higher. Similarly, under the unbalanced data condition, the scatter plots (e-h) show a gradual improvement in model performance with the progressive addition of each component.

### SHAP analysis

**Fig 10:**
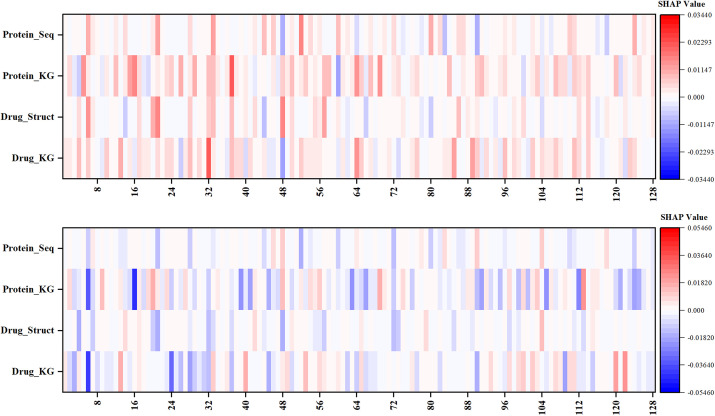
SHAP heatmaps for a positive (top) and a negative (bottom) sample across different feature modalities.

In this section, SHapley Additive exPlanations (SHAP) is employed to elucidate the model’s decision-making process. Specifically, one positive and one negative sample were randomly selected, and their SHAP values were calculated across 128 feature dimensions from different modalities, as illustrated in Fig 10. In the heatmaps, color intensity indicates the magnitude of each feature’s contribution to the final prediction, with red denoting positive contributions and blue representing negative ones. In the positive sample, the majority of feature dimensions across all modalities exhibit positive SHAP values, with several dimensions demonstrating particularly significant contributions—suggesting that the model successfully identifies discriminative information supporting the positive prediction. In contrast, the heatmap for the negative sample is predominantly blue, suggesting that the feature dimensions collectively guide the model toward a negative decision. Notably, both heatmaps contain dimensions with near-zero contributions and even opposing effects, implying that the prediction emerges from a dynamic trade-off among diverse features rather than being driven by a few dominant signals.

A cross-modality comparison further reveals that the KG modality consistently exhibits higher contribution scores, highlighting its dominant role in the prediction. Meanwhile, the structural/sequence modality, though less prominent, still offers complementary information that supports the final fusion-based decision. These findings demonstrate that the proposed model is capable of effectively identifying and leveraging discriminative features across modalities to differentiate between positive and negative samples.

### Case study: 5-HT2A

To demonstrate the exceptional predictive capability of KG-MACNF, we select 5-HT2A (UniProtKB Entry: P28223) as the target of interest for our case study. Table 7 presents the top ten drug candidates predicted by KG-MACNF for this target. To validate these predictions, we consult the Drug-Target Interactions Checker tool provided by PubMed and DrugBank. For example, we predict that Sertindole has a 0.99 probability of interacting with 5-HT2A, a finding supported by the literature (PMID: 16925508). The study reports that Sertindole, an antipsychotic drug, has affinity for dopamine D2, 5-HT2A, 5-HT2C serotonin receptors, and α-1 adrenergic receptors. This further confirms that KG-MACNF serves as a promising new tool for drug discovery and development.

**Table 7:** Top ten drugs that interact with 5-hydroxytryptamine receptor 2A.

DrugBank ID	Drug Name	Prediction Score	Actions	Evidence
DB14011	Cannabinoids	0.999	Unknow	PMC4604182
DB01621	Pipotiazine	0.999	Antagonist	PMID: 10821438
DB06144	Sertindole	0.999	Antagonist	PMID: 16925508
DB06016	Cariprazine	0.999	Antagonist	PMID: 26510944
DB00409	Remoxipride	0.999	Unknow	PMID: 9015795
DB01151	Desipramine	0.999	Antagonist	PMID: 7855217
DB01623	Thiothixene	0.998	Antagonist	PMID: 10821438
DB00363	Clozapine	0.998	Antagonist	PMID: 12973385
DB00420	Promazine	0.998	Antagonist	PMID: 10980325
DB08810	Cinitapride	0.998	Antagonist	PMID: 9211565

## Discussion

The KG-MACNF model has achieved notable advancements in DTI prediction. However, several limitations remain, and further research is needed to improve its performance and applicability. One potential limitation lies in the use of random negative sampling, which may introduce false negatives and weaken the model’s discriminative ability. More refined strategies—such as hard negative mining based on molecular or structural similarity, or heuristic sampling guided by domain-specific knowledge—could be explored to generate more informative and biologically meaningful negative instances. In addition, while KG-MACNF shows competitive performance in cold-start settings, its generalization to completely unseen drugs or targets remains challenging. This highlights the potential value of incorporating pretrained molecular and protein language models to derive transferable sequence-level embeddings, thereby enhancing the model’s robustness in out-of-distribution scenarios. Another promising direction is to situate DTI prediction within a broader multi-task learning framework involving related tasks such as drug-drug interaction (DDI), compound-protein interaction (CPI), and protein-protein interaction (PPI). Such integration could enable cross-task representation sharing and relational knowledge transfer, ultimately enhancing the generalization ability of the model. Furthermore, improving model interpretability remains an important direction. While this study utilizes SHAP-based analysis to enhance transparency, future work could explore progressive, in-training interpretability techniques to better align explanation with the learning dynamics. For instance, KAN can progressively visualizing and analyzing its learned B-spline basis functions throughout the training process, which may provide insights into how complex drug–target interactions are encoded as training progresses. Finally, considering the evolving nature of biomedical knowledge, the development of online learning algorithms capable of real-time updates is essential. In parallel, developing a user-friendly software interface could further enhance the usability of KG-MACNF, promoting its adoption in real-world drug discovery workflows.

## Conclusion

The proposed KG-MACNF model integrates multi-relational embeddings of drug and target entities within biological networks and employs multi-head cross-modal attention along with KDFN nonlinear mapping to deeply combine various features, thereby enhancing the accuracy of DTI prediction. Experimental results on two publicly available datasets demonstrate that KG-MACNF outperforms 13 baseline methods in terms of AUC and AUPR. In cold start scenarios, the model effectively overcomes the limitations of single-modal data when drug or protein information is missing by integrating multi-modal information (i.e., structural sequence features of biomolecules and multi-relational data from biological KG), thereby ensuring the stability and accuracy of predictions. Notably, even under scenarios with highly imbalanced distributions of positive and negative samples, KG-MACNF exhibits remarkable discriminative power. Furthermore, an ablation study confirms that simple feature concatenation is insufficient to fully leverage the advantages of different modality features in DTI task. By emphasizing critical modality-specific information and minimizing noise interference, KG-MACNF achieves significant improvements in predictive performance. The SHAP-based interpretability analysis further reveals the relative contributions of different modalities to the prediction task, while clearly illustrating the decision-making rationale of the model. A case study further illustrates that KG-MACNF can accurately identify drug candidates for specific targets, with validation supported by relevant literature. Overall, by addressing the shortcomings of previous models in utilizing modality information and feature complementarity, KG-MACNF provides a more reliable predictive tool for drug discovery.
